# MosaicBase: A Knowledgebase of Postzygotic Mosaic Variants in Noncancer Disease-related and Healthy Human Individuals

**DOI:** 10.1016/j.gpb.2020.05.002

**Published:** 2020-09-08

**Authors:** Xiaoxu Yang, Changhong Yang, Xianing Zheng, Luoxing Xiong, Yutian Tao, Meng Wang, Adam Yongxin Ye, Qixi Wu, Yanmei Dou, Junyu Luo, Liping Wei, August Yue Huang

**Affiliations:** 1 Center for Bioinformatics, State Key Laboratory of Protein and Plant Gene Research, School of Life Sciences, Peking University, Beijing 100871, China; 2 Department of Bioinformatics, Chongqing Medical University, Chongqing 400016, China; 3 College of Life Sciences, Beijing Normal University, Beijing 100875, China; 4 National Institute of Biological Sciences, Beijing 102206, China; 5 Peking-Tsinghua Center for Life Sciences (CLS), Academy for Advanced Interdisciplinary Studies, Peking University, Beijing 100871, China; 6 Chinese Academy of Medical Sciences and Peking Union Medical College, Beijing 100730, China; 7 School of Life Sciences, Peking University, Beijing 100871, China

**Keywords:** Postzygotic, Mosaicism, Noncancer, Mutation, MosaicBase

## Abstract

Mosaic variants resulting from **postzygotic** mutations are prevalent in the human genome and play important roles in human diseases. However, except for cancer-related variants, there is no collection of postzygotic mosaic variants in **noncancer** disease-related and healthy individuals. Here, we present **MosaicBase**, a comprehensive database that includes 6698 mosaic variants related to 266 noncancer diseases and 27,991 mosaic variants identified in 422 healthy individuals. Genomic and phenotypic information of each variant was manually extracted and curated from 383 publications. MosaicBase supports the query of variants with Online Mendelian Inheritance in Man (OMIM) entries, genomic coordinates, gene symbols, or Entrez IDs. We also provide an integrated genome browser for users to easily access mosaic variants and their related annotations for any genomic region. By analyzing the variants collected in MosaicBase, we find that mosaic variants that directly contribute to disease phenotype show features distinct from those of variants in individuals with mild or no phenotypes, in terms of their genomic distribution, **mutation** signatures, and fraction of mutant cells. MosaicBase will not only assist clinicians in genetic counseling and diagnosis but also provide a useful resource to understand the genomic baseline of postzygotic mutations in the general human population. MosaicBase is publicly available at http://mosaicbase.com/ or http://49.4.21.8:8000.

## Introduction

Genomic mosaicism results from postzygotic mutations that arise during embryonic development, tissue self-renewal [1], aging processes [2], or exposure to other DNA-damaging circumstances [3]. Unlike *de novo* or inherited germline variants that affect every cell in the carrier individual [4], postzygotic mosaic variants only affect a portion of cells or cell populations, and their mutant allelic fractions (MAFs) should be less than 50% [5]. If a postzygotic mutation affects germ cells [6], the mutant allele may theoretically be transmitted to offspring, which is the major source of genetic variations in the human population [7].

Postzygotic mosaic variants have previously been demonstrated to be directly responsible for the etiology of cancer [8], [9] and an increasing number of other Mendelian or complex diseases, including epilepsy-related neurodevelopment disorders [10], Costello syndrome [11], autism spectrum disorders [12], [13], and intellectual disability [14]. On the other hand, pathogenic genetic variants inherited from detectable parental mosaicism have been demonstrated to be an important source of monogenic genetic disorders, including Noonan syndrome [15], Marfan syndrome [16], Dravet syndrome [17], and complex disorders, including autism [18] and intellectual disability [19]. The MAF of a mosaic variant has been reported to be directly related to the carrier’s phenotype [20], [21] and to be associated with the recurrence risk in children [5].

With the rapid advances in next-generation sequencing (NGS) technologies, tens of thousands of postzygotic mosaic single-nucleotide variants (SNVs) and insertions/deletions (indels) have been identified in the genomes of human individuals [3], [22], [23]. However, except for cancer-related variants that have been collected by databases such as the Catalogue of Somatic Mutations in Cancer (COSMIC) [24] and somatic mutations impacting microRNA function in cancer (SomamiR) [25], there is no integrated database focusing on mosaic variants in noncancer disease-related and healthy individuals.

Here, we present MosaicBase. To our best knowledge, MosaicBase is the first knowledgebase of mosaic SNVs and indels identified in patients with noncancer diseases and their parents or grandparents as well as healthy individuals. MosaicBase currently contains 34,689 validated mosaic variants that have been manually curated from 383 publications. MosaicBase has further integrated comprehensive genomic and phenotypic information about each variant and its carrier. It provides multi-scale information about disease-related mosaic variants for genetic counseling and molecular diagnosis, as well as the genomic background of mosaic variants in general population.

## Database implementation

### Framework of MosaicBase

**Figure 1:**
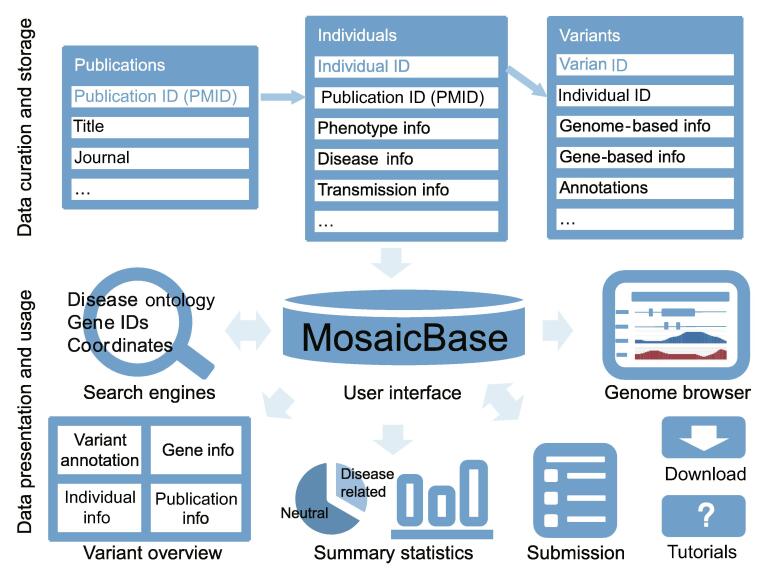
**Overview of the data collection, storage, and visualization of MosaicBase**

An overview of the framework of MosaicBase is shown in Figure 1. MosaicBase consists of two logical parts: the database and server as the backend, and the user interface as the frontend. Structured data based on three main relational tables were established in the backend of MosaicBase. The storage and maintenance of the database were implemented with SQLite v3. The frontend of MosaicBase provides a user-friendly interface written in Personal home page Hypertext Preprocessor (PHP), JavaScript, HyperText Markup Language (HTML), and Cascading Style Sheets (CSS), with Django applications.

MosaicBase incorporates two different search modes (basic mode and ontology-based mode) to help users browse the database. The information for each mosaic variant has been summarized from the publication and individual levels to the gene and variant levels. A built-in genome browser is provided to visualize variants. The statistical summaries and detailed tutorials for MosaicBase are available on the main page. MosaicBase further provides an online submission system to encourage the community to contribute to the database.

### Data collection, processing, and annotation

We queried against the PubMed database using keywords including “mosaic”, “mosaicism”, “post-zygotic”, “somatic”, “sequencing” (see the full query string in File S1), and excluded publications about cancer-related mosaic mutations or studies on non-human organisms by examining the titles and abstracts. For more than 1000 search results, we scrutinized the main text as well as supplemental information to confirm the relevance of each publication. After this process, 383 journal research articles about mosaic SNVs and indels in noncancer individuals that were published between Jan 1989 and May 2018 were collected into MosaicBase. For each article, data fields for the publication, individual, and variation information were extracted and saved into tables in the backend (Figure 1). For studies involving single-cell technologies, only the validated or high-confidence postzygotic mosaic SNVs were collected. For the table of variation information, we further integrated genomic annotations generated by ANNOtate VARiation (ANNOVAR) [26], including population allele frequency from dbSNP (version 137) [27] and gnomAD (genome; version 2.0.1) [28], risk scores such as Combined Annotation-Dependent Depletion (CADD) scores (version 1.30) [29] and Eigen scores [30], functional predictions by Functional Analysis through Hidden Markov Models (FATHMM) [31], Sorting Intolerant From Tolerant (SIFT) [32], iFish2 [33], DeFine [34], conservation prediction by GERP++ [35] and PhyloP [36], and annotations in COSMIC [37]. A detailed description of different fields and data types required in each field is listed in Tables S1, S2, and S3. The transcript-based variation information was confirmed using Mutalyzer following the suggestions from the Human Genome Variation Society (HGVS) [38]. Genomic coordinates were provided according to the human reference genome from University of California at Santa Cruz (UCSC) hg19/GRCh37 as well as hg38/GRCh38.

### Statistical analysis and visualization of mosaic variants

Mutation signature analysis has been widely used in cancer studies to elaborate the etiology of somatic mosaic variants, by decomposing the matrix of tri-nucleotide context into cancer-related signatures. In this study, the signature of noncancer mosaic variants was analyzed by Mutalisk [39], and the maximum likelihood estimation of proportions for each mutation signature was performed based on a greedy algorithm. For each variant group, we further tested whether its genomic density within each 1-Mb interval was linearly correlated with the GC content, DNase I hypersensitive (DHS) regions, replication timing, and histone modification profiles measured in the GM12878 cell line [39]. A genome browser based on the Dalliance platform [40] was implemented to interactively visualize mosaic variants. Circos [41] was utilized to show the genomic distribution of mosaic variants.

## Web interface

### User interface and functions

**Figure 2:**
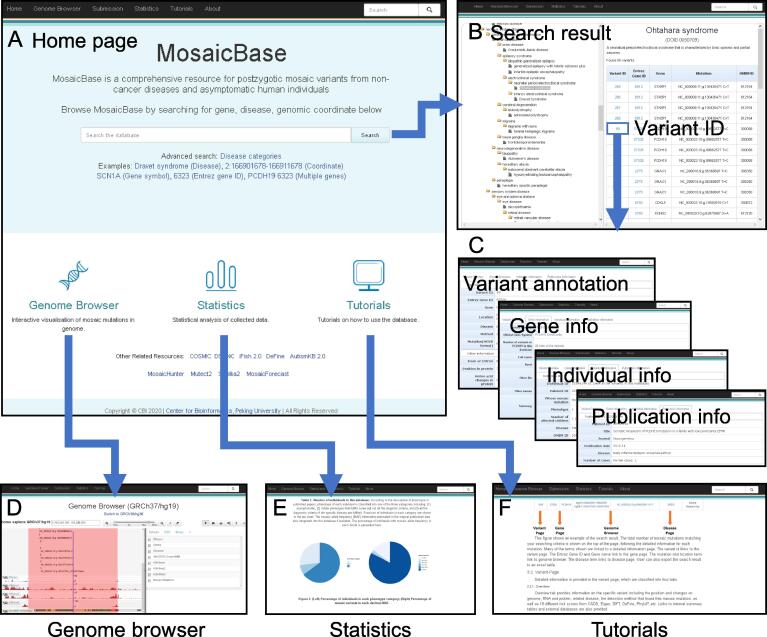
**Screenshots of MosaicBase** **A.** The main page provides the search modes and multiple links to different utilities of the database. **B.** Disease ontology-based advanced search page and an example of a result table. **C.** The variant pages from the basic search results; these pages provide information about each variant and its corresponding gene, individual and publication annotation, the individuals carrying the same variant, as well as the publications describing the variant. **D.** Integrated genome browser to visualize mosaic variants with genetic and epigenetic annotations. **E.** Summary statistics of the publications, mutational spectrum, and individuals collected in MosaicBase. **F.** Detailed tutorials for the introduction, data presentation, and usage of MosaicBase.

We incorporate two search modes in MosaicBase. The basic search mode provided on the main page recognizes search terms based on the name of diseases, the range of genomic coordinates, gene symbols, or Entrez Gene IDs (Figure 2A), in which the search engine is comparable with space-delimited multiple search terms. The result page of the basic search mode displays variant summary information according to the categories of search terms, and search results can then be downloaded as an .xls format table. We also include an ontology-based search mode as an advanced option in MosaicBase. With this mode, users can browse the mosaic variants relevant to a specific disease or disease category according to the Disease Ontology [42]. A brief summary of the description of the disease or disease category is provided along with a summary table of all the related mosaic variants collected in MosaicBase (Figure 2B).

Detailed information about each mosaic variant is summarized in four different panels in MosaicBase. These include the overview panel, the gene information panel, the individual information panel, and the publication information panel (Figure 2C). In the overview panel, we provide the genomic information as well as the identification and validation methodologies for each variant. In the gene information panel, we annotate each gene by its Entrez Gene ID, official gene symbol and alternative names, number of reported mosaic variants in this gene, Vega ID, OMIM ID, Human Genome Organisation Gene Nomenclature Committee (HGNC) ID, Ensembl ID, and a brief gene summary. In addition, we also summarize all the collected mosaic variants from the same gene and provide links for gene annotation from external databases. In the individual information panel, we classify the phenotypes of the individual carrying the mosaic variant and display the information according to the original descriptions in the publication. The severity of phenotype collected in MosaicBase is defined as “1” if the carrier was healthy, “2” if the carrier had a mild phenotype but did not fulfill all the diagnostic criteria for a specific disease or characterized syndrome, and “3” if the carrier fulfilled all the clinical diagnostic criteria for a specific disease. In the publication information panel, we summarize the title, journal, sample, and additional information about the publication which reports the mosaic variant.

MosaicBase integrates a build-in genome browser to provide convenient interactive data visualization for the mosaic variants (Figure 2D). In addition to the built-in tracks about genetic and epigenetic annotations, such as DHS sites and H3K4me predictions, MosaicBase also allows the user to import customized tracks from URLs, UCSC-style track hubs, or to upload files in a UCSC-style genome browser track format. The URLs for tracks of Ensembl Gene and methylated DNA immunoprecipitation sequencing (MeDIP-seq) data are provided as examples, and a help page providing detailed guidance is also available by clicking the question mark in the top-left panel of the genome browser. MosaicBase further provides users with an application that can generate publication-quality scalable vector graphic (SVG) files from the control panel of the genome browser.

MosaicBase included a “Statistics” page to show a summary of all the collected mosaic variants (Figure 2E) and a “Tutorials” page (Figure 2F) with detailed introductions about the database, as well as its search modes, data presentation, and genome browser. We also implement an online submission system that allows users to submit mosaic variants from newly published or uncollected publications. Such variants will be manually examined by our team and integrated into MosaicBase with scheduled updates.

### Statistical analysis of noncancer mosaic variants

**Figure 3:**
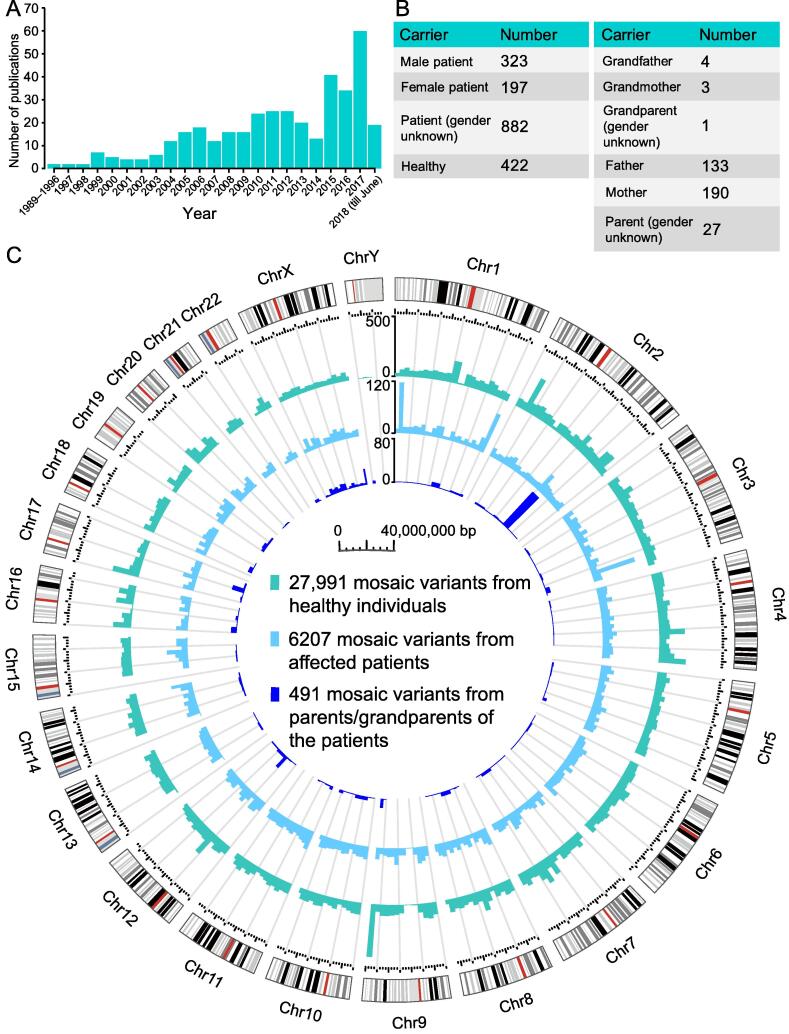
**Statistics about the publication, individual, and variant data collected in MosaicBase** **A.** Number of relevant publications from 1989 to June 2018 collected from PubMed. Data before 1997 were condensed. Query terms and inclusion/exclusion criteria for literature acquisition are described in File S1. **B.** Summary of different categories of mosaic carriers. Left: Number of patients (grouped by gender) and healthy individuals. Right: Number of parents and grandparents (grouped by gender). **C.** Genomic distribution of mosaic variants. Chromosomal bands are illustrated in the outer circle with centromeres in red. Histograms show the count of mosaic variants for 1-Mb interval in each inner circle. Genomic coordinates and color codes of the categories are shown in the center.

MosaicBase currently includes 383 journal research articles, letters, and clinical genetic reports about noncancer postzygotic mosaic variants that were published from 1989 to 2018 (Figure 3A), with an accelerated accumulation of relevant publications boosted by the recent advances in NGS technologies. After manually extracting the mosaic variants reported in each publication, we thoroughly compiled 34,689 mosaic variants from 2182 human individuals, including 6698 disease-related variants from 3638 genes related to 266 noncancer diseases from 1402 patients and 358 parents or grandparents of the patients, as well as 27,991 apparently neutral variants identified from 422 healthy individuals (Figure 3B and Table S4). Specifically, two types of disease-related mosaic variants have been collected in MosaicBase: (1) 6207 mosaic variants that directly contribute to the disease phenotype in 1402 patients (323 men, 197 women, and 882 cases with gender information not available from the original publications); and (2) 491 mosaic variants identified from 358 parents or grandparents (137 men, 193 women, and 28 cases with gender information not available from the original publications) of the probands who had transmitted the mosaic allele to their offspring for a heterozygous genotype that lead to disease phenotypes (Figure 3B). The collected mosaic variants are classified into three groups according to the origin of the variants described in the original publications. Variants from healthy individuals are termed the “healthy” group; variants from patients fulfilling the full diagnostic criteria of a specific disease are termed the “patients” group; and variants from parents/grandparents of the patients are termed the “parents/grandparents” group. As shown in Figure 3C, mosaic variants are generally distributed across all the autosomes and X chromosomes. Parental mosaic variants are clustered in the *SCN1A* gene on chromosome 2, which results from the well-studied parental mosaic cases for Dravet syndrome [17], [20]. The underrepresentation of mosaic variants in the Y chromosome might be explained by its low gene density and the technical challenge of detecting mosaic variants in haplotype chromosomes.

**Figure 4:**
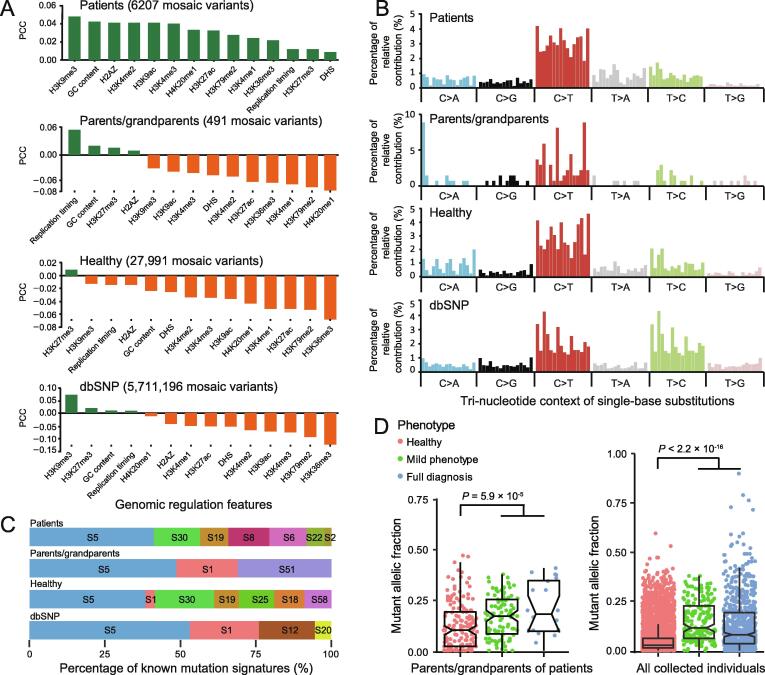
**Genomic features of mosaic variants collected in MosaicBase** **A.** PCC of the density of mosaic variants and genomic features. Genomic density within each 1-Mb interval was linearly correlated with GC content, DHS regions, replication timing, and histone modification profiles measured in the GM12878 cell line. **B.** Tri-nucleotide genomic context of mosaic variants. **C.** Estimated contribution of mutation signatures for mosaic variants. 60 single-base substitution signatures and artefact signatures from COSMIC were considered, and a linear regression model was used to estimate the proportion of signatures. **D.** Mutant allele fraction of 491 mosaic variants in 358 individuals from parents/grandparents group (left) and 34,689 variants in all the 2182 individuals (including 1402 noncancer patients, 358 parents/grandparents of the patients, and 422 healthy individuals) collected in MosaicBase (right). Significantly higher mosaic allele fractions were observed in individuals with disease phenotypes (*P* = 5.9 × 10^−5^ and *P* < 2.2 × 10^−16^; two-tailed Mann–Whitney *U* test). In panels A–C, 5,711,196 common germline variants with population allele frequency ≥ 0.1 in dbSNP (version 137) were shown for comparison. H2AZ, histone 2A.Z variant; DHS, DNase I hypersensitive; PCC, Pearson correlation coefficient.

To study whether mosaic variants from different groups of individuals have distinct genomic characteristics, we calculated their correlation with various genomic regulation features, including GC content, DHS positions, and epigenetic modifications. Because the vast majority of mosaic variants have been identified from peripheral blood or saliva samples, genomic regulation annotations of the lymphoblastoid cell line GM12878 was used in this analysis. Common germline variants annotated in dbSNP 137 with allele frequency higher than 10% (“dbSNP” group) serve as a control. According to the Pearson correlation coefficients between the signal intensities of genomic features and the density of variants with a window size of 1 Mb across the genome [43], we find that the mosaic variants contributing directly to the disease phenotype (“patients” group) are more positively correlated with such genomic features than the mosaic variants of other groups (Figure 4A).

Next, we examined the mutation spectrum of the collected mosaic variants. Similar to inherited germline variants [44] and somatic variants reported in cancer studies [45], C > T is the most predominant type for mosaic variants (Figure 4B). We then extracted the tri-nucleotide genomic context of each variant and decomposed the matrix into mutation signatures previously identified in various types of cancers (https://cancer.sanger.ac.uk/cosmic/signatures). Mutation signature analysis further revealed that over 50% of the mosaic variants can be decomposed into the combination of cancer signatures 1, 5, and 30, whereas the remaining mosaic variants consist of signatures 2, 6, 8, 12, 18, 19, 20, 22, 25, 51, and 58 (Figure 4C). Signatures 1 and 5 result from the age-related process of spontaneous or enzymatic deamination of 5-methylcytosine to thymine; signatures 18 and 30 result from deficient base excision repair [46]; signature 2 indicates the activation of AID/APOBEC cytidine deaminase; signatures 6 and 20 are associated with defective DNA mismatch repair; signature 22 is associated with aristolochic acid exposure. The etiology of signatures 8, 12, 19, 25 are unknown, whereas signatures 51 and 58 are potential sequencing artefacts. Detailed descriptions of the signatures are provided in File S1.

To explore the general relationship between the MAF of a mosaic variant and its carrier’s phenotype, we extracted the allele fraction and phenotypic severity information for each mosaic variant in MosaicBase. Among parents and grandparents of patients (“parents/grandparents” group), we observed that the mosaic variants from carriers manifesting mild or full disease phenotypes had significantly higher MAFs than those from carriers without any disease phenotypes (*P* = 5.9 × 10^−5^; two-tailed Mann–Whitney *U* test with continuity correction, Figure 4D), which is in accordance with previous observations [18], [20], [47]. When we considered mosaic variants in all the collected individuals, the difference became even more significant (*P* < 2.2 × 10^−16^; two-tailed Mann–Whitney *U* test, Figure 4D). These results highlight the importance of the MAF information of mosaic variants in clinical applications such as genetic counseling.

## Discussion

MosaicBase currently contains 34,689 mosaic SNVs and indels identified from patients with noncancer diseases and their parents or grandparents, as well as from healthy individuals, with rich information at the publication, individual, gene, and variant levels. The user-friendly interface of allows users to access MosaicBase by multiple searching methods and the integrated genome browser.

The pathogenic contribution of mosaic variants to noncancer diseases has been increasingly recognized in the past few years. MosaicBase provides genetic and phenotypic information about 6698 disease-related mosaic variants in 266 noncancer diseases. This database may help clinicians understand the pathogenesis and inheritance of mosaic variants and shed new light on future clinical applications, such as genetic counseling and diagnosis. On the other hand, the collection of 27,991 mosaic variants identified in healthy individuals could be useful for understanding the genomic baseline of postzygotic mutations in the general human population. MosaicBase also integrates risk prediction from multiple computational tools for each variant. Unlike germline variants which are present in all cells of the carriers, mosaic variants are only present in a fraction of cells, in which the level of mosaic fraction can be an additional factor contributing to variant pathogenicity [18], [20]. In the future, with the increasing number of relevant studies, we would expect a well-benchmarked scoring system specifically designed for predicting the deleterious probability of mosaic variants.

Of the 34,689 mosaic variants collected in MosaicBase, only 0.7%–8.7% are present in large-scale population polymorphism databases (Table S5). If we only consider common SNPs with population allele frequency (AF) higher than 0.01, the overlapping proportion further reduces to 0.1%–0.7%. This suggests that MosaicBase provides a unique set of human genetic variants which have been overlooked in previous genomic studies. Indeed, these apparently benign variants that are generated *de novo* show characteristics distinct from those of the variants that directly contribute to a disease phenotype, and also different from polymorphisms that are fixed in population under selective pressure (Figure 4). The data collected by MosaicBase will encourage researchers to reanalyze existing NGS data of human diseases by mosaic variant calling tools, such as MosaicHunter [48], Mutect2 [49], and Strelka [50], to identify previously ignored disease causative variants.

In the future, our team will update MosaicBase regularly by collecting and reviewing new publications in PubMed and publications submitted through our online submission system. After each update, we will update the statistics and release update reports on the website. We plan to further improve the user interface of MosaicBase and add new analysis tools based on feedback from the community.

## Data availability

MosaicBase is publicly available at http://mosaicbase.com/ or http://49.4.21.8:8000.

## CRediT author statement

**Xiaoxu Yang:** Conceptualization, Data curation, Formal analysis, Software, Methodology, Visualization, Writing - original draft, Writing - review & editing, Project administration. **Changhong Yang:** Data curation, Software, Methodology, Formal analysis, Methodology, Visualization, Writing - original draft. **Xianing Zheng:** Data curation, Software, Methodology, Formal analysis, Visualization, Writing - original draft. **Luoxing Xiong:** Data curation. **Yutian Tao:** Data curation. **Meng Wang:** Software. **Adam Yongxin Ye:** Software. **Qixi Wu:** Data curation, Supervision. **Yanmei Dou:** Data curation. **Junyu Luo:** Data curation. **Liping Wei:** Conceptualization, Resources, Writing - review & editing, Funding acquisition, Supervision. **August Yue Huang:** Conceptualization, Supervision, Software, Writing - original draft, Writing - review & editing, Supervision, Project administration. All authors read and approved the final manuscript.

## Competing interests

The authors declare no competing interests.
