# Supplementary material for: MosaicBase: A Knowledgebase of Postzygotic Mosaic Variants in Noncancer Disease-related and Healthy Human Individuals
Source: Genomics Proteomics Bioinformatics. 2020 Sep 8;18(2):140–9. doi: 10.1016/j.gpb.2020.05.002 (PMC7646124; doi:10.1016/j.gpb.2020.05.002)
Supplement: Supplementary data 1 [file mmc1.doc]

**File S1 Data collection and mutational signatures**

**Literature curation and variant collection**


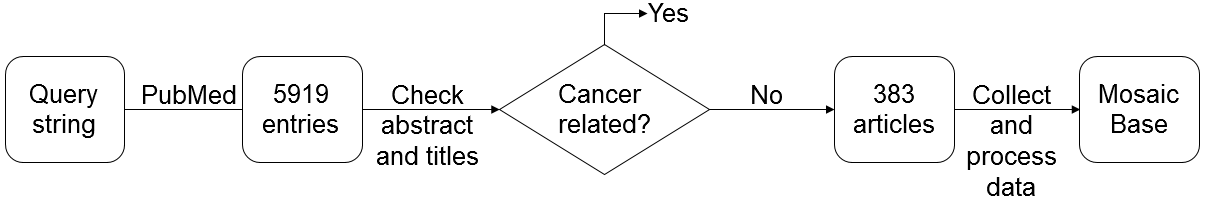


The query string for PubMed was “((mosaic[Title/Abstract] OR mosaicism[Title/Abstract] OR neurogenesis[Title/Abstract] OR (post zygotic)[Title/Abstract] OR somatic[Title/Abstract] ) AND ((next generation sequencing) OR (deep sequencing) OR (sequencing) OR mutational) NOT cancer[Title] NOT tumor[Title/Abstract] NOT tumour[Title/Abstract] NOT plant NOT leukaemia NOT *oma NOT virus NOT transgen* NOT knockout NOT knockin NOT (carcinoma) NOT (sarcoma) NOT review[Publication Type]) AND ("1989/01/01"[Date - Publication] : "2018/06/01"[Date - Publication])”, and a total of 5919 results were returned. We further excluded all publications about cancer-related mosaic mutations or studies on non-human organisms by manual check the title and abstract. For the remaining publications, we scrutinized the main text as well as supplemental information to further confirm their relevance to our study. As a result, 383 journal research articles passed all the filters. We manually collected detailed information at publication-, individual-, and variant-level. Genomic coordinates of each variant were computed from their cDNA accession using Mutalyzer [1] or directly obtained from the publication, and further converted between hg19/RCh37 and hg38/GRCh38 version via UCSC liftover.

**Detail description of single-base substitution signatures**

For any single-base substitution, there are only six different possible substitutions: C>A, C>G, C>T, T>A, T>C, and T>G. Considering the nucleotide context, 4 conditions in the +1 and -1 genomic position, there are at most 96 conditions. A signle-base substitution signature is generated by presenting the relative contribution of the 96 different conditions in a single figure for any given collection of variants. Almost all the commonly used mutation signatures for mosaic mutations are identified from the collections of the ICGC/TCGA Pan Caner Analysis of Whole Genomes Network [2, 3]. The stability and reproducibility of these signatures were assessed from 1865 whole genomes and 19,148 exomes.

*Signature 1*

Single-base substitution signature 1 (SBS1/S1) represents an endogenous mutational process initiated by spontaneous or enzymatic deamination of 5-methylcytosine to thymine which generates G:T mismatches in double-stranded DNA. Failure to detect and remove these mismatches prior to DNA replication results in fixation of the T substitution for C. S1 is clock-like in that the number of mutations in most cancers and normal cells correlates with the age of the individual. Rates of acquisition of S1 mutations over time differ markedly between different cancer types and different normal cell types. These differences correlate with estimated rates of stem cell division in different tissues and S1 may therefore be a cell division/mitotic clock.


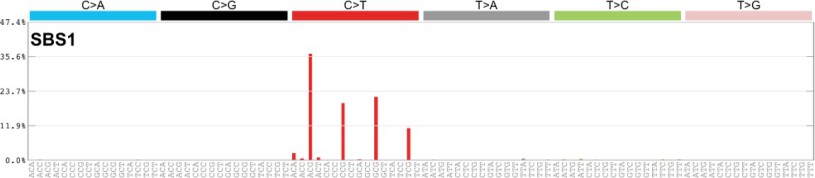


*Signature 2*

SBS2/S2 is usually found in the same samples as S13. It has been proposed that activation of AID/APOBEC cytidine deaminases in cancer may be due to previous viral infection, retrotransposon jumping, or tissue inflammation.


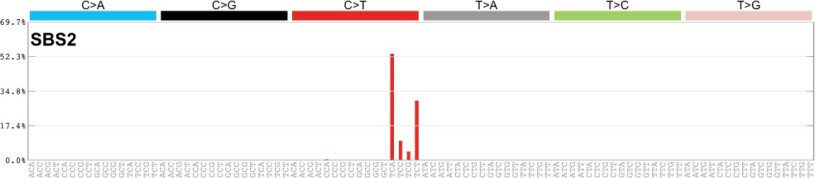


*Signature 5*

SBS5/S5 is clock-like in that the number of mutations in most cancers and normal cells correlates with the age of the individual. Rates of acquisition of S5 mutations over time differ between different cancer types and different normal cell types. These differences do not clearly correlate with estimated rates of stem cell division in different tissues nor with differences in S1 mutation rates. S5 may be contaminated by S16.


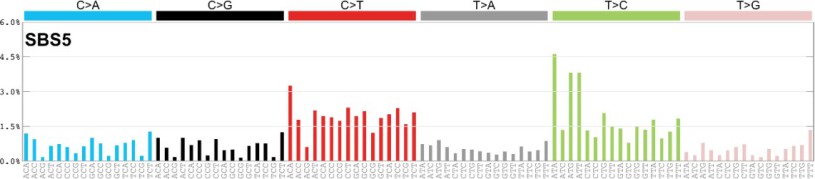


*Signature 6*

SBS6/S6 is one of seven mutational signatures associated with defective DNA mismatch repair (with microsatellite instability, MSI) and is often found in the same samples as other MSI-associated signatures: S14, S15, S20, S21, S26, and S44.


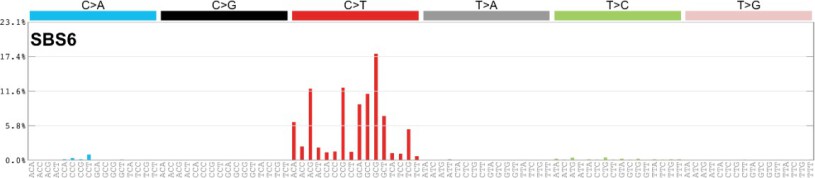


*Signature 8*

The etiology of SBS8/S8 is unknown, it is associated with CC > AA mutations.


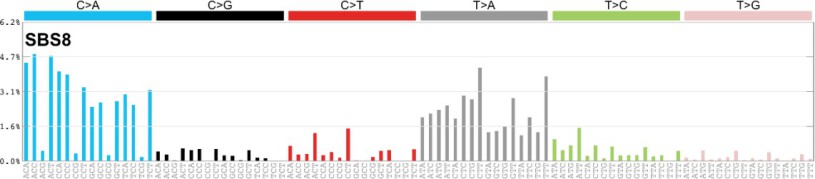


*Signature 12*

The etiology of SBS12/S12 is unknown.


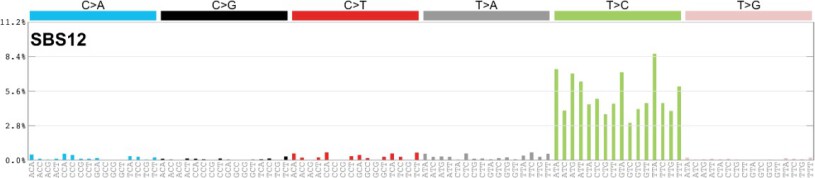


*Signature 18*

SBS18/S18 is similar in profile to S36, which is associated with defective base excision repair.


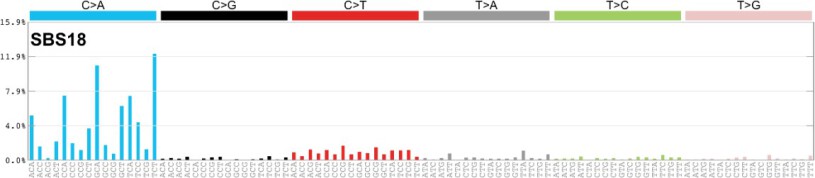


*Signature 19*

The etiology of SBS19/S19 is unknown.


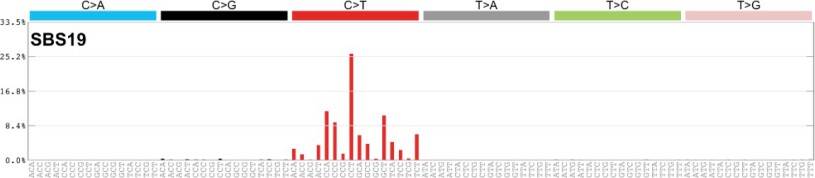


*Signature 20*

SBS20/S20 is one of seven mutational signatures associated with defective DNA mismatch repair (MSI) and is often found in the same samples as other MSI-associated signatures: S6, S14, S15, S21, S26, and S44.


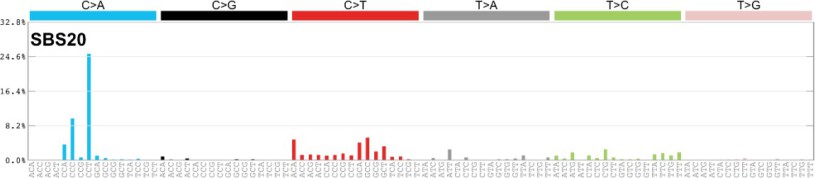


*Signature 22*

SBS22/S22 has been found in experimental systems exposed to aristolochic acid.


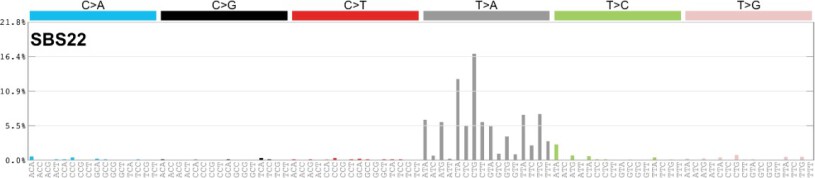


*Signature 25*

The etiology of SBS25/S25 is unknown. This signature was identified in Hodgkin’s cell lines.


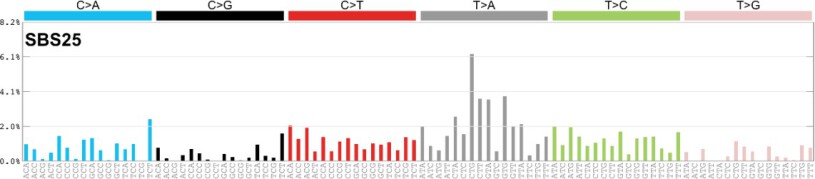


*Signature 30*

SBS30/S30 is due to deficiency in base excision repair.


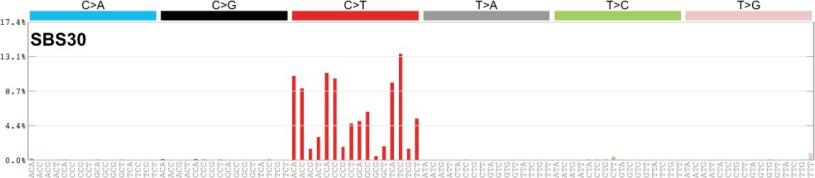


*Signature 51*

SBS51/S51 is a potential sequencing artefact.


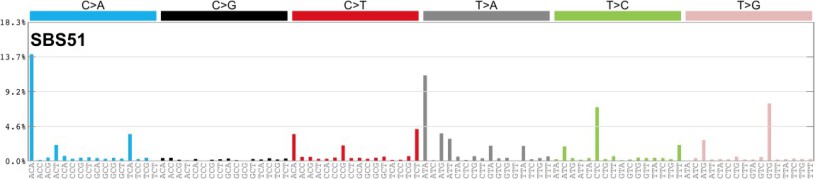


*Signature 58*

SBS58/S58 is a potential sequencing artefact.


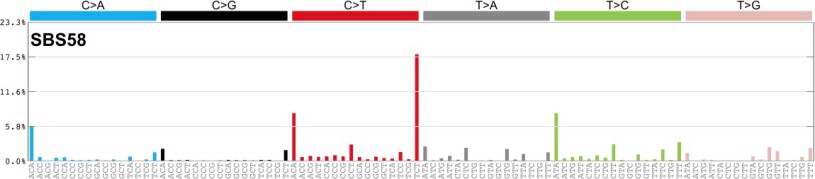


**Web resources**

MosaicBase: http://mosaicbase.com/ or http://49.4.21.8:8000/

Biodalliance: http://www.biodalliance.org/

Disease Ontology: http://disease-ontology.org

Mutalisk: http://mutalisk.org/

Mutalyzer: https://mutalyzer.nl/

**References**

[1] Wildeman M, van Ophuizen E, den Dunnen JT, Taschner PE. Improving sequence variant descriptions in mutation databases and literature using the Mutalyzer sequence variation nomenclature checker. Hum Mutat 2008;29:6–13.

[2] Alexandrov LB, Nik-Zainal S, Wedge DC, Aparicio SA, Behjati S, Biankin AV, et al. Signatures of mutational processes in human cancer. Nature 2013;500:415–21.

[3] Alexandrov LB, Kim J, Haradhvala NJ, Huang MN, Tian Ng AW, Wu Y, et al. The repertoire of mutational signatures in human cancer. Nature 2020;578:94–101.
