# Supplementary material for: MosaicBase: A Knowledgebase of Postzygotic Mosaic Variants in Noncancer Disease-related and Healthy Human Individuals
Source: Genomics Proteomics Bioinformatics. 2020 Sep 8;18(2):140–9. doi: 10.1016/j.gpb.2020.05.002 (PMC7646124; doi:10.1016/j.gpb.2020.05.002)
Supplement: Supplementary data 2 [file mmc2.docx]

**Table S1 Field description for the table of publication information**

| **Field name** | **Description** | **Required or optional** |
| --- | --- | --- |
| PMID | Accession number of the publication in PubMed | Required |
| Title | Title of publication | Required |
| Journal | Journal of publication | Required |
| Publication_date | Date of acceptation | Required |
| Disease | Name of the disease related to the publication | Required |
| OMIM | Accession number of the disease in OMIM | Required |
| Population | Population of the cohort studied | Optional |
| Incidence_lower | Lower bound of the incidence rate of the disease | Optional |
| Incidence_higher | Upper bound of the incidence rate of the disease | Optional |
| Male_case | Number of male patients carrying mosaic variants | Optional |
| Female_case | Number of female patients carrying mosaic variants | Optional |
| Case | Number of patients carrying mosaic variants | Required |
| Age_effect | Does the disease have age effect (paternal/maternal) | Required |
| Notes | Additional notes | Optional |
