# Supplementary material for: MosaicBase: A Knowledgebase of Postzygotic Mosaic Variants in Noncancer Disease-related and Healthy Human Individuals
Source: Genomics Proteomics Bioinformatics. 2020 Sep 8;18(2):140–9. doi: 10.1016/j.gpb.2020.05.002 (PMC7646124; doi:10.1016/j.gpb.2020.05.002)
Supplement: Supplementary data 3 [file mmc3.docx]

**Table S2**  **Field description for the table of individual information**

| **Field name** | **Description** | **Required or optional** |
| --- | --- | --- |
| PMID | Accession number of the publication in PubMed | Required |
| Individual_ID | ID of the individual in MosaicBase (described as PubMed ID + 01/02/03/04) | Required |
| Whose_mosaic | The individual who is carrying the mosaicism | Required |
| Patient_mutation_origin | Parent-of-origin for a patient with mosaic variant | Optional |
| Phenotype_mosaic | Severity of the carrier of mosaic mutation (1: asymptomatic; 2: mildly affected; 3: severely affected; 4: matching all the criteria of a specific disease) | Optional |
| Age_lower | Lower bond of age | Optional |
| Age_upper | Upper bond of age | Optional |
| Affected_child_nc | Number of affected children of the individual | Optional |
| Affected_male_child_nc | Number of affected sons of the individual | Optional |
| Affected_female_child_nc | Number of affected daughters of the individual | Optional |
| Affected_grandson | Number of affected grandsons of the individual | Optional |
| Affected_granddaughter | Number of affected granddaughters of the individual | Optional |
| Disease | Name of non-cancer disease of the individual | Required |
| OMIM | Accession number of the disease in OMIM | Required |

*Note*: nc, number of counts.
