# Supplementary material for: MosaicBase: A Knowledgebase of Postzygotic Mosaic Variants in Noncancer Disease-related and Healthy Human Individuals
Source: Genomics Proteomics Bioinformatics. 2020 Sep 8;18(2):140–9. doi: 10.1016/j.gpb.2020.05.002 (PMC7646124; doi:10.1016/j.gpb.2020.05.002)
Supplement: Supplementary data 4 [file mmc4.docx]

| **Table S3**  **Field description for the table of variation information** | | |  |
| --- | --- | --- | --- |
| **Field name** | **Description** | **Required or optional** | **PMID** |
| Individual_ID | ID of the individual in MosaicBase (described as PubMed ID + 01/02/03/04) | Required |  |
| Variation_ID | ID of the variant in MosaicBase (described as Individual ID + 01/02/03/04) | Required |  |
| Gene | Name of variant if located on an annotated gene | Required |  |
| Chromosome | The chromosome where the variant is located (in hg19) | Required |  |
| Position_start | The start position of the variant on genome (in hg19) | Required |  |
| Position_end | The end position of the variant on genome (in hg19, likely equal to position end) | Required |  |
| DNA_Ref_nt | Reference DNA sequence of the variant | Required |  |
| DNA_Alt_nt | Alternative DNA sequence of the variant | Required |  |
| Genome_assembly | Version of the reference genome assembly | Required |  |
| Exon_or_ intron | Whether the variant is located in exon or intron | Required |  |
| Exon_number | Exon number if the variant is located on an exon | Optional |  |
| Exon_nc | Number of nucleotides from the exon if the variant is located on an exon | Optional |  |
| Protein_position | Position of the variant on the protein sequence | Required |  |
| Ref_aa | Reference amino acid of the variant | Required |  |
| Alt_aa | Altered amino acid of the variant | Required |  |
| Frameshift | If the variant is a frameshift variant | Optional |  |
| aa_indel | Whether there are indels in the amino acid sequence | Optional |  |
| cDNA position | Position of the variant on the cDNA sequence | Required |  |
| Ref_nt | Reference nucleotide sequence of the variant from cDNA | Required |  |
| Alt_nt | Alternative nucleotide sequence of the variant | Required |  |
| nt_indel | insertion deletion | Required |  |
| mRNA_accession | Accession No. of the cDNA sequence | Required |  |
| mRNA_length | Length of the cDNA sequence | Required |  |
| Relative_loc_gene | Relative location on the gene: percentage of the cDNA position of the variant over the cDNA full length | Required |  |
| Genome_length (Mb) | Length of the chromosome according to reference genome | Required |  |
| Relative_loc_genome | Percentage of the genomic position of the variant over the full length of the chromosome | Required |  |
| Mosaic_allelic_fraction_lower | Lower bound of the mosaic allelic fraction | Optional |  |
| Mosaic_allelic_fraction_upper | Upper bound of the mosaic allelic fraction | Optional |  |
| Total_read | Total read coverage of the validation/identification of the variant if detected by NGS | Optional |  |
| Sample_type | Type of sample (blood, bone, skin, saliva, *etc*.) | Optional |  |
| Quantification_method | Method for quantifying the mosaic variant (Roche454, Sanger sequencing, Illumina Hiseq2000, *etc*.) | Optional |  |
| Note | Additional information | Optional |  |
| hg38_chr_pos1_pos2 | The genomic coordinates of the variant in hg38 | Required |  |
| gnomAD_MAF | The population allele frequency of the variant in the Genome Aggregation Database version 2.0.1 | Optional | 32461654 |
| dbSNP137 | The SNP ID in dbSNP version 137 | Optional | 11125122 |
| COSMIC_ID | The variant ID in the Catalogue of Somatic Mutations in Cancer, COSMIC version 89 | Optional | 27727438 |
| COSMIC_occurrence | The variant occurrences in COSMIC version 89 | Optional | 27727438 |
| Eigen | The Eigen score of functional importance | Optional | 26727659 |
| CADD13_raw | The raw score by the Combined Annotation-Dependent Depletion, CADD version 1.3 | Optional | 24487276 |
| CADD13_PHRED | The prediction score of CADD version 1.3, deleterious if the score is > 10, tolerated if the score is <= 10 | Optional | 24487276 |
| FATHMM | The raw prediction score by the Functional Analysis Through Hidden Markov Models, FATHMM, deleterious if the score is < −1.5, tolerated if the score is >= −1.5 | Optional | 23033316 |
| SIFT_score | Prediction score by the Sorting Intolerant From Tolerant method, SIFT | Optiona | 12824425 |
| SIFT_pred | Prediction result by SIFT, D for deleterious and T for tolerated | Optional | 12824425 |
| LRT_score | The Likelihood Ration Test, LRT score | Optional | 26555599 |
| LRT_pred | Score predicted by LRT, D for deleterious, N for Neutral, and U for unknown | Optional | 26555599 |
| MutationTaster_score | Score by MutationTaster | Optional | 20676075 |
| MutationTaster_pred | Prediction by MutationTaster, D for manually annotated disease causing, A for automatically annotated disease causing, N for manually annotated polymorphism, and P for automatically annotated polymorphism | Optional | 20676075 |
| MutationAssessor_score | Score by MutationAssessor | Optional | 17976239 |
| MutationAssessor_pred | Predictions by MutationAssessor, H and M for functional, L and N for non-functional | Optional | 17976239 |
| PROVEAN score | Score predicted by PROVEAN | Optional | 25851949 |
| PROVEAN_pred | Prediction result by PROVEAN, D for deleterious, and N for neutral | Optional | 25851949 |
| MetaSVM_score | Score by the meta-analytic Support Vector Machine, MetaSVM | Optional | 28149325 |
| MetaSVM_pred | Prediction result by MetaSVM, D for deleterious and N for neutral | Optional | 28149325 |
| MetaLR_score | Score by MetaLR | Optional | 28149325 |
| MetaLR_pred | Prediction result by MetaLR, D for deleterious and T for tolerated | Optional | 28149325 |
| M-CAP_score | Score by the Mendelian Clinically Applicable Pathogenicity score, M-CAP | Optional | 27776117 |
| M-CAP_pred | Prediction result by M-CAP, D for deleterious and T for tolerated | Optional | 27776117 |
| Integrated_fitCons_score | Score by fitCons | Optional | 23749186 |
| Integrated_confidence_value | Significance in fitCons score, highly significant for *P* < 0.003, significant for *P* < 0.05, informative for *P* < 0.25, and other for *P* >=0.25 | Optional | 23749186 |
| GERP++_RS | Score for the Genomic Evolutionary Rate Profiling updated score | Optional | 21152010 |
| phyloP100way_vertebrate | Phylogenetic *P* values, phyloP score based on multiple alignment of 100 vertebrates | Optional | 19858363 |
| phastCons100way_vertebrate | The PHylogenetic Analysis with Space/Time models, phastCons score based on multiple alignment of 100 vertebrates | Optional | 16024819 |
| SiPhy_29way_logOdds | The Site-specific PHYlogenetic analysis, SiPhy log transformed odds ratio on multiple alignment of 29 mammals | Optional | 19478016 |
| iFish2_probability | Deleterious probability by the integrated functional inference of SNVs in human, iFish, version2 | Optional | 27527004 |
| iFish2_pred | Prediction by iFish2 | Optional | 27527004 |
| DeFine_score | Deleterious probability by the deep learning based functional impact of non-coding variants evaluator, DeFine | Optional | 29617928 |
| DeFine_Pred | Prediction by DeFine | Optional | 29617928 |

*Note*: nc, number of counts.
