# Supplementary material for: MosaicBase: A Knowledgebase of Postzygotic Mosaic Variants in Noncancer Disease-related and Healthy Human Individuals
Source: Genomics Proteomics Bioinformatics. 2020 Sep 8;18(2):140–9. doi: 10.1016/j.gpb.2020.05.002 (PMC7646124; doi:10.1016/j.gpb.2020.05.002)
Supplement: Supplementary data 5 [file mmc5.docx]

**Table S4 Summary for mosaic SNVs and indels in noncancer disease-related and healthy individuals in MosaicBase**

|  | | **Noncancer disease-related individuals** | **Healthy individuals** | **Total** |
| --- | --- | --- | --- | --- |
| Variants | Number of variants in genic regions (number of genes) | 6658 (3638) | 15,168 (8131) | 21,826 (9256) |
|  | Number of variants in intergenic regions | 40 | 12,823 | 12,863 |
|  | Total number of variants | 6698 | 27,991 | 34,689 |
| Individuals | Number of parents/grandparents | 358 | - | 2182 |
|  | Number of patients | 1402 | - |  |
|  | Number of healthy individuals | - | 422 |  |
| Number of publications | | 377 | 14 | 383 |
| Number of noncancer diseases | | 266 | - | 266 |

*Note*: All the raw data including the disease and variant information are available at <http://49.4.21.8:8000/media/data/MosaicBase_Full_Data.xls>.
