# Supplementary material for: MosaicBase: A Knowledgebase of Postzygotic Mosaic Variants in Noncancer Disease-related and Healthy Human Individuals
Source: Genomics Proteomics Bioinformatics. 2020 Sep 8;18(2):140–9. doi: 10.1016/j.gpb.2020.05.002 (PMC7646124; doi:10.1016/j.gpb.2020.05.002)
Supplement: Supplementary data 6 [file mmc6.docx]

**Table S5 Comparisons between postzygotic mosaic variants collected in MosaicBase and human genetic variations identified by large-scale sequencing projects**

| **Dataset** | **Description** | **Total** | **Overlap with MosaicBase** | **Overlap with MosaicBase and with population AF ≥ 0.01** |
| --- | --- | --- | --- | --- |
| ExAC (r0.3.1) | The Exome Aggregation Consortium | 14.9 million | 1004 | 27 |
| ESP6500 | The NHLBI GO Exome Sequencing Project | 1.1 million | 235 | 0 |
| UK10K | The UK10K Project | 24 million | 693 | 135 |
| 1KGP (phase 3) | The 1000 Genomes Project | 84.4 million | 1308 | 231 |
| dbSNP (version 137) | A database of single nucleotide polymorphisms | 192.7 million | 1074 | 213 |
| gnomAD: genome (version 2.0.1) | The Genome Aggregation Database | 229.9 million | 3028 | 243 |

*Note:* AF, allele frequency.
